# Mesenchymal Stem Cell-Derived Extracellular Vesicle Therapy for Stroke: Challenges and Progress

**DOI:** 10.3389/fneur.2019.00211

**Published:** 2019-03-12

**Authors:** Oh Young Bang, Eun Hee Kim

**Affiliations:** ^1^Department of Neurology, Samsung Medical Center, Sungkyunkwan University School of Medicine, Seoul, South Korea; ^2^Translational and Stem Cell Research Laboratory on Stroke, Samsung Medical Center, Seoul, South Korea; ^3^Medical Research Institute, Sungkyunkwan University School of Medicine, Seoul, South Korea; ^4^Stem cell and Regenerative Medicine Institute, Samsung Biomedical Research Institute, Seoul, South Korea

**Keywords:** stroke, ischemic stroke, extracellular vesicles, stem cells, mesenchymal stem cells, microRNA

## Abstract

Stroke is the leading cause of physical disability among adults. Stem cells such as mesenchymal stem cells (MSCs) secrete a variety of bioactive substances, including trophic factors and extracellular vesicles (EVs), into the injured brain, which may be associated with enhanced neurogenesis, angiogenesis, and neuroprotection. EVs are circular membrane fragments (30 nm−1 μm) that are shed from the cell surface and harbor proteins, microRNAs, etc. Since 2013 when it was first reported that intravenous application of MSC-derived EVs in a stroke rat model improved neurological outcomes and increased angiogenesis and neurogenesis, many preclinical studies have shown that stem cell-derived EVs can be used in stroke therapy, as an alternative approach to stem cell infusion. Although scientific research regarding MSC-derived EV therapeutics is still at an early stage, research is rapidly increasing and is demonstrating a promising approach for patients with severe stroke. MSC therapies have already been tested in preclinical studies and clinical trials, and EV-mediated therapy has unique advantages over cell therapies in stroke patients, in terms of biodistribution (overcoming the first pass effect and crossing the blood-brain-barrier), cell-free paradigm (avoidance of cell-related problems such as tumor formation and infarcts caused by vascular occlusion), whilst offering an off-the-shelf approach for acute ischemic stroke. Recently, advances have been made in the understanding of the function and biogenesis of EVs and EVs therapeutics for various diseases. This review presents the most recent advances in MSC-derived EV therapy for stroke, focusing on the application of this strategy for stroke patients.

## Introduction

Stroke is the leading cause of physical disability among adults. One-fourth to a half of stroke survivors are left with significant disabilities. Stem cell therapy is considered a potential regenerative strategy for patients with neurologic deficits. Adult stem cells, such as mesenchymal stem cells (MSCs) may be a good option for stroke therapy, as they secrete a variety of bioactive substances, including trophic factors and extracellular vesicles (EVs, 30 nm−1 μm sized circular membrane fragments shed from the cell surface) into the injured brain, which is associated with enhanced neurogenesis, angiogenesis, and synaptogenesis (1–5). In addition, MSCs are thought to play multiple roles, such as attenuating inflammation (6), reducing scar thickness (7), enhancing autophagy (8), and possibly replacing damaged cells (9), in various brain diseases. Over the past 15 years, several randomized stem cell therapy trials have been conducted in patients with ischemic stroke (10–17), which showed mixed results. Possible reasons for conflicting results include, heterogeneous study populations (therefore requiring the selection of optimal candidate patients), delayed treatment (thus requiring an off-the shelf approach as soon as possible following a stroke), the limited restorative potential of stem cell therapy (especially in elderly patients with chronic illness), and a lack of objective measurements for the assessment of efficacy in stem cell therapy (5, 18).

It is widely accepted that MSCs exert their action via paracrine effects via secretomes or EVs, rather than through transdifferentiation to replace damaged neurons. Approximately 80% of cells disappeared in the infarcted brain within several days after transplantation with MSCs (19), yet the effects of stem cells persisted for several weeks following treatment. Our biodistribution study showed that MSCs exhibit a dynamic release of EVs in the ischemic brain condition, and that systemic administration of MSC-derived EVs led to a dose-dependent increase of MSC EVs in the infarcted hemisphere (bypassing the lung and liver) and functional improvement, suggesting that MSC EV therapy has a similar functional outcome, yet an improved safety profile compared to MSC administration (20).

This review presents the most recent advances in MSC-derived EV therapy for stroke, focusing on the clinical application of this strategy for stroke patients.

## Biology and Function of Extracellular Vesicles

### EV Biogenesis

EVs are a broad term that usually refers to heterogeneous vesicles that are released from cells. EVs containing cellular proteins, DNAs and RNAs of cells are classified into exosomes (30–200 nm), microvesicles (200–1000 nm) and apoptosomes (1–10 μm) depending on their size (21). Among them, exosomes and microvesicles released from living cells, are involved in many processes, such as proliferation, differentiation and angiogenesis, and are known to act as a means of intercellular communication (22–24).

**Figure 1:**
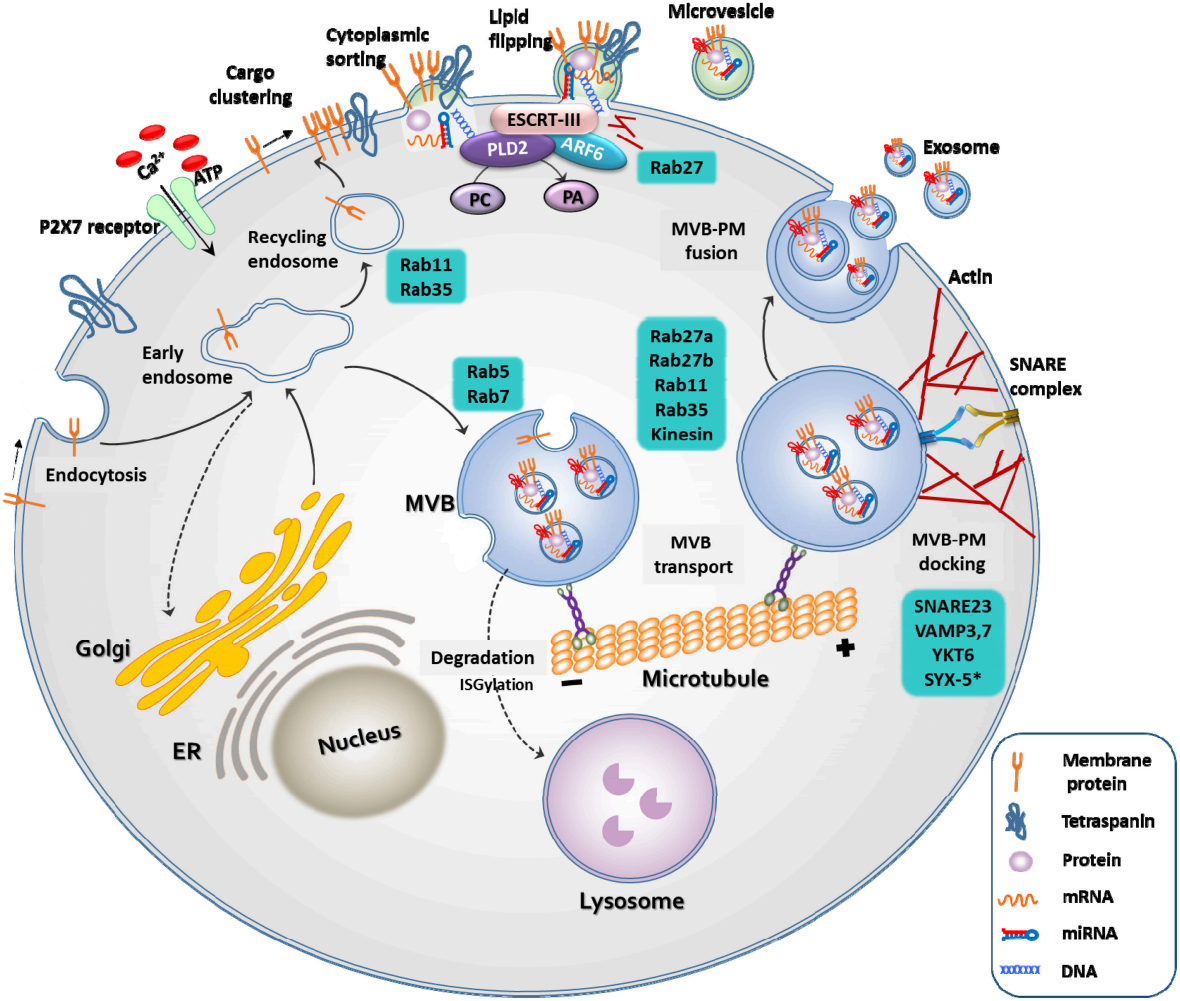
Biogenesis of extracellular vesicles. EVs are released through two different pathways. When extracellular adenosine triphosphate (ATP) increases in response to external stimuli, the P2X7 receptor opens and calcium ions enter the cell. Membrane-associated proteins, tetraspanins, and cytoplasmic cargos are clustered in discrete membrane of the plasma membrane for microvesicles. The cargo of MVs are composed of cytoplasmic proteins, mRNAs, miRNAs, and DNAs. Similar to exosomes, RAS-related protein (RAB), actin, the endosomal sorting complex required for transport (ESCRT), ADP ribosylation factor 6 (ARF6) and phospholipase D2 (PLD2), and soluble N-ethylmaleimide-sensitive protein receptor (SNARE) proteins play important roles in MV release. However, MVs differ from exosomes in that they bud directly through flipping of lipid from the plasma membrane. The cargo of multivesicular bodies (MVBs) are either derived from endocytosis of the plasma membrane or from the trans-Golgi network. The reverse flow in the direction of the Golgi or recirculation to the plasma membrane is controlled by various Rab GTPases. Once MVB has matured, it is transported to the plasma membrane along the microtubule, and not by lysosomes. As a final step in exosome release, MVBs are docked and fused with the plasma membrane. Rab, actin, and SNARE proteins play important roles in these exosome release steps.

Exosomes and microvesicles originate from the plasma membrane, and are formed through distinct mechanisms (Figure 1) (23). The generation of microvesicles begins with the recruitment of cytoplasmic proteins and nucleic acids by the endosomal sorting complex required for transport (ESCRT)-dependent and independent pathways {mediated by ADP ribosylation factor 6 [ARF6] and phospholipase D2 [PLD2]. Lipid flipping then occurs, and membrane budding takes place.

Microvesicles are a more heterogeneous population and more sensitive to external stimulation than exosomes. For example, an increase in the extracellular concentration of ATP induces activation of the P2X7 receptor and consequential release of microvesicles (21). The production of exosomes begins with the membrane folding inward, the creation of empty intraluminal vesicles (ILVs), and the maturation of ILVs into multivesicular bodies (MVBs). They are released into the extracellular space through fusion of MVBs and the plasma membrane by small GTPases, such as RAB27A, RAB11, and RAB35, or by ESCRT (25).

Knowledge regarding EV biogenesis is essential for understanding EV characteristics and for the development of EV therapeutics. For example, activation of P2X7R by the pathogen-associated molecular pattern (PAMP) or damage-associated molecular pattern (DAMP) can induce membrane blebbing, or fusion of MVBs. PLD2, which regulates lipids by degrading phosphatidylcholine into choline and phosphatidic acid, and ARF6, which regulates membrane trafficking and actin cytoskeleton remodeling, may play an important role in endocytosis and exocytosis (23). Studies have shown that overexpression of ARF6 increases the number of exosomes released from the cells, whereas inhibition of ARF6 and PLD2 reduces the release of exosomes (26, 27).

### Mechanisms of Action of Stem Cell-Derived EVs

Stem cell-derived EVs could play a critical role in the exchange of information between stem cells and damaged cells and alter the behavior of the target cells. Ischemia induces an increase in the circulating or regional levels of EVs, and it has been identified that EVs have their own function. Stroke triggers the mobilization of bone marrow (BM) MSC-derived EVs in patients with severe stroke (28), and EVs released by ischemic stimulation have restorative capacity (20). In addition, EVs from ischemic tissue facilitated vasculogenesis in the ischemic limb model (29). EVs from ischemic muscles induce BM mononuclear cell differentiation into cells with an endothelial phenotype (29).

Formation of new neuronal cells and blood vessels are the fundamental processes for the recovery after ischemic brain injury. During acute phase of stoke, both ischemic/reperfusion injury and inflammatory response are pivotal to the pathophysiology of ischemic stroke. In addition, stroke patients are often elderly and have chronic diseases, which may attenuate regenerative potential after stroke. As shown in Table 1, regenerative potential could be enhanced by treatment of MSCs or MSC-derived EVs (67). In addition, treatment of MSC-derived EVs in animal models of brain diseases resulted in central and peripheral immunotolerance (63, 68). EVs harbor bioactive molecules and EVs secreted from stem cells carry more complex cargos than other cellular sources (69). Stem cell-derived EVs contain many molecules that may have therapeutic effects in stroke (70), such as microRNAs, proteins, and mitochondria (Table 1). MicroRNAs, are a class of short, single-stranded, non-coding RNAs that can be horizontally shuttled by EVs, and EVs-encapsulated proteins have been implicated in the regulation of protective and restorative processes (71). Beside microRNAs, MSC EVs may shuttle other genetic components, such as mRNAs (72). In addition, damage to the mitochondria caused by tissue injury, aggravates the severity of injury. Restoration of mitochondria dysfunction, through stem cell-derived mitochondria transplantation via EVs could potentially be an effective therapeutic strategy (66, 73).

**Table 1:** Mode of action of stem cell-derived EV in animal models of stroke or other ischemic disease.

**Mode of action**	**Intravesicular contents**
Angio-/neuro-genesis	miR-17-92 cluster targeting phosphatase and tensin homolog (30) miR-124a (31) miR-126 targeting portocadherin 7 (32) miR-133b targeting RABEPK (33) and RhoA (34) miR-134 targeting caspase-8 (35) miR-181b-5p targeting TRPM7 (36) miR-184 targeting Numbl, miR-210 targeting ephrin-A3 (20, 37, 38) miR-210 targeting Efna3 (39) miR-294 (40) Angiopoietin-1 mRNA to restore vascular permeability (41) CXCR4 via Akt signaling pathway (42) CXCR4, VEGF, VEGFR2, HGF, c-Met, Akt (20) VEGF, HIF-1α (43) PDGF (44) ICAM-1, bFGF, CHI3L1, CD147, CD105 (37) Transcription factors (STAT3) and signaling pathways (NF-κB) (45, 46) No specified (47–49)
Neuroprotection	miR-19a targeting PTEN (50) miR-21 via MAPK signaling pathway (51) miR-22 targeting Mecp2 (52) miR-125b targeting p53 (53) miR-145 targeting AQP4 (54) miR-199a via sirt1 pathway (55) miR-214 targeting CaMKII (56) miR-494 via Akt pathway (57) miR-711 targeting PPARγ (58) Neuron-specific enolase (59) Plasminogen activator inhibitor-1 targeting STAT3 and Akt (60)
Immunomodulation	miR-181a via BCL2, XIAP (61) CD73 promote adenosine accumulation (62) Anti-inflammatory cytokines (63) Not specified (64)
Rejuvenation	miR-17, 34a via Akt signaling (65) Mitochondria (66)
Thrombus resolution and recanalization	miR-126 targeting portocadherin 7 (32)

*miR, microRNA; RABEPK, Rab9 effector protein with kelch motifs; RhoA, Ras homolog gene family member A; TRPM7, transient recent potential melastatin 7; NF-κB, nuclear factor-κB; PDGF, platelet-derived growth factor*.

## Advantages of Extracellualr Vesicles Over Stem Cells in Stroke

Allogeneic stem cells have many advantages over autologous stem cells. Allogeneic MSCs are scalable from a manufacturing perspective, with standardized procedures. The use of allogeneic MSCs reduces the time required to obtain a sufficient number of cells (the “off the shelf” approach). Through the application of allogeneic stem cell therapy in the acute phase of stroke, both neurorestorative and neuroprotective actions can be expected (74). In recent clinical trials of intravenous application of allogeneic stem cells (MultiStem® in patients with acute stroke, stem cells were applied within 24–48 h, following the onset of symptoms (16). In addition, MSCs from younger healthy donors may differ in terms of their proliferation and neurorestorative capacity, from those obtained from elderly stroke patients with chronic illness (75).

However, conflicting results exist. Following serum contact, allogeneic MSCs can be injured by complement, and the viability of allogeneic MSCs after infusion is greatly reduced, compared with autologous MSCs (76). High mortality following intravenous transplantation of MSCs in animal stroke models, and reports of pulmonary embolism following intravenous injection of allogeneic adipose-derived MSCs have been accounted (77). MSC-related procoagulation status could be a possible explanation for such lethal pulmonary thromboembolism (78). Lastly, cell diameters of MSCs are large, ranging from 15 to 30 μm, which leads to passive arrest of MSCs in small diameter vessels, causing vascular occlusion and reduction in cerebral blood flow, when administered through intra-arterial routes, and also trapping in systemic vessels such as the lungs, when administered systemically (the first pass effect) (79–81).

The cell-free paradigm, using allogeneic MSC-derived EVs could avoid such cell-related problems of allogeneic stem cell therapy. EVs have low toxicity, high stability in the circulation, advantages in scalable production and storage, and high transport efficiency to donor cells (passing the blood-brain barrier [BBB] and avoiding the first pass effect).

## Applications of Stem Cell-Extracellular Vesicles for Treating Stroke

### Preclinical Evidence of the Effects of EVs Derived From Various Stem Cells in Stroke

Xin et al. reported for the first time, that intravenous application of MSC-derived EVs in a stroke rat model improved neurological outcomes and increased angiogenesis and neurogenesis (47). Other investigators have also demonstrated that stem cell-derived EVs can be used for stroke therapy, as an alternative approach to stem cell infusion methods (Table 2) (67, 87, 88). In addition, several advances in EV-based strategy were introduced, including: (a) the use of stem cells other than MSCs, such as EVs from embryonic stem cells (ESCs), neural stem cells (NSCs), or induced pluripotent stem cells (iPSC)-derived MSC/NSCs (64, 84, 86), (b) application of EVs via the intra-nasal approach (84), (c) EV production other than conventional two-dimensional (2D) culture methods to increase the production of EVs and regulate the contents of EVs, e.g., 3D dynamic culture (37) and stimulation with ischemic brain extracts (20, 59), and (d) various EV isolation methods other than ultracentrifugation (82, 83, 85). Very recently, the effects of EVs on stroke has been tested in a large animal model of stroke (86).

**Table 2:** Various applications of stem cell-derived EV in stroke.

**References**	**Animals**	**Stem cells** **/mode of application**	**EV production** **/culture media**	**EV isolation** **/dose per animal**	**Major finding**
Xin et al. (47)	Rat	Rat BM MSCs /intravenous	2D culture /Exosome-free serum	UC /100 μg total exosome protein	Angiogenesis Neurogenesis Neurological recovery
Doeppner et al. (82)	Mice	Human BM MSCs /intravenous	2D culture /MSC basal media	PEG /EVs released by 2 × 10^6^ cells	Neuroprotection Angiogenesis Neurogenesis Immunomodulation Neurological recovery
Chen et al. (83)	Rat	Mini-pig adipose MSCs /intravenous	2D culture /10% fetal bovine serum	KISO™ system /100 μg total exosome protein	Reduction of infarct volume Neurological recovery
Lee et al. (59)	Rat	Human adipose MSCs /intravenous	2D culture /Serum free media with brain extract	UC /0.2 mg /kg	Angiogenesis Neurogenesis Immunomodulation
Kalani et al. (84)	Mice	Mice ESCs /intra-nasal	2D culture on fibroblast monolayer /Exosome-free serum	UC /NA	Restoration of neurovascular unit Immunomodulation
Otero-Ortega et al. (85)	Rat	Rat adipose MSCs /intravenous	2D culture /Exosome-free serum	Exosome extraction kit (miRCURY) /100 μg total exosome protein	Neuroplasticity White matter recovery Neurological recovery
Xin et al. (30)	Rat	Rat BM MSCs /intravenous	2D culture /Exosome-free serum	UC /100 μg total exosome protein	Neuroplasticity Neurological recovery
Xin et al. (33)	Rat	microRNA-133b overexpressing Rat BM MSCs /intra-arterial	2D culture /Exosome-free serum	UC /3 × 10^11^ EVs, comparable to 100 μg total exosome protein	Neuroplasticity Neurological recovery
Moon et al. (20)	Rat	Rat BM MSCs /intravenous	2D culture /Serum free media with brain extract	UC /30 μg total exosome protein	Angiogenesis Neurogenesis Neuroplasticity Neurological recovery
Cha et al. (37)	*In vitro*	Human BM MSCs	3D dynamic culture /Serum free media	UC /NA	Angiogenesis Neurogenesis Neurological recovery
Webb et al. (86)	Pig	Human NSCs /intravenous	2D culture /NSC basal culture media	UC /2 × 10^10^ EVs /kg	Improve neural tissue preservation Neurological recovery
Webb et al. (64)	Mice	iPSC-derived NSC or MSCs /intravenous	NA	NA	Neuroprotection Immunomodulation Neurological recovery

*MSC, mesenchymal stem cells; BM, bone marrow; UC, ultracentrifugation; PEG, polyethylene glycol precipitation method; ESCs, embryonic stem cells; iPSC, induced pluripotent stem cell; NSCs, neural stem cells; NA, not available*.

### Recent Advances for EV Therapeutics

Various approaches are currently being employed to drive the MSC secretome toward a more anti-inflammatory and regenerative phenotype (88). Because secretomes include a wide array of growth factors, cytokines, and EVs, such approaches could also improve the efficacy of EV-based therapy.

Firstly, conventional 2D cell culture systems often disregard the mechanical stimuli that significantly influence the intricate *in vivo* cellular microenvironment. Characteristics of EVs as well as phenotypes of stem cells could be affected by mechanical forces (89). For example, shear stress enhances the immune regulatory function of MSCs (90). In addition, compared to conventional 2D cultured MSCs, MSCs cultured in spheroid showed higher efficacy and safety profiles, and decreased the expression of integrins, resulting in increased secretion of EVs (91, 92). Cha et al. successfully amplified EV sections and therapeutic EV contents (microRNAs and cytokines) from MSCs using a dynamic 3D culture method, instead of using the conventional culture method (37). In a traumatic brain injury model, EVs derived from MSCs cultured in 3D scaffolds provided better outcomes than EVs from MSCs cultured in 2D conditions, probably by promoting neurogenesis and angiogenesis (93). Either native (decellularizing tissues) or synthetic 3D extracellular matrix-based scaffolds can be utilized to provide a 3D environment for cell attachment and growth (23).

Second, although MSC-derived EVs show promise in their application for regenerative therapies, their use is often limited by very low-yield conventional cell culture systems. Both microcarriers and hollow-fiber bioreactors are currently used for large-scale cell expansion of MSCs in the 3D environment (23) (89). These methods may be particularly useful in MSC EV production, because (a) large volumes of media would be required to get a sizable number of EVs for clinical use, (b) viability of MSCs could be maintained by continuous medium perfusion and avoiding metabolic by-product accumulation in a bioreactor, without the use of serum, which contains a large number of xenogeneic EVs, and (c) continuous processing, by controlling culture medium flow in and out of a bioreactor, as is often required because of the high advantages of reproducibility and safety of the resulting EV products.

Third, preconditioning of sublethal stimuli can trigger an adaptive response to further injury or damage. A wide variety of molecules and culture methods can be used to prime MSCs and modify their EVs. For example, Moon et al. showed that cultivation of MSCs with either serum obtained from stroke patients, or treatment of ischemic brain extracts on culture media, could activate restorative properties of MSCs and the release of EVs, suggesting that signals from an ischemic brain can affect the efficacy of MSCs and MSC-derived EVs and activate the secretion of EVs from MSCs (20, 94). Similar findings were also reported by another research group (59). It is widely accepted that hypoxic conditions (i.e., 0.1–2% O_2_, conditions similar to BM) were beneficial to MSCs and might stimulate MSCs to exhibit adaptive responses. MSC culture in hypoxic conditions with/without serum deprivation amplified EV sections, increased therapeutic EV contents (e.g., microRNAs), and improved the EV efficacy in tissue-injury models (48, 49, 56, 95). Inflammatory stimulation of MSCs renders release of EVs that have enhanced anti-inflammatory properties (96).

Fourth, as mentioned before, there have been advances in our current knowledge on the regulation of EV biogenesis (Figure 1). The modification of certain molecular pathways in EV biogenesis could lead to increased yield of EV production (23). For example, activation of EV biogenesis during membrane blebbing (P2X7 receptor, phospholipase D2) or multivesicular body fusion with the plasma membrane (Rab GTPase, SNARES) could increase EV secretion, leading to an increased yield (23, 25, 97–100). In addition, genetic modification to overexpress certain therapeutic proteins or RNAs within EVs (Table 2) could lead to an increased efficacy of EVs. For example, EVs harvested from microRNA-133b-overexpressing MSCs improved neuronal plasticity and functional recovery following stroke (33). Furthermore, bioengineering techniques can be applied to produce semi-synthetic artificial EVs to increase the expression of functional/traceable molecules on EV surfaces/membranes or cargo, and fully synthetic artificial EVs can be engineered to increase the yield of EV production (101). For example, “exosome-like nanovesicles,” which have morphological and biochemical characteristics similar to EVs, can be made from cells through cell membrane fragmentation (102).

Lastly, the source of EVs could be an important determinant in the efficacy of stem cell-derived EVs in stroke. MSCs have limited restorative potential in elderly patients. Similarly, MSC EVs may have significant age-dependent differences in their cargo contents (103). The transfer of EVs from young MSCs rejuvenated aged stem cells (65). Fetal MSCs from amniotic fluid, cord blood, or Wharton's Jelly-derived stem cells are reported to have intermediate cellular phenotypes between ESCs/iPSC and MSC, in terms of expression patterns of both marker/transcription factors of pluripotency and mesenchymal commitment, as well as their broadly multipotent nature (104). Although the use of ESC/iPSC-derived EV therapy may be safer than the use of ESC/iPSC cell therapy, in terms of tumorigenicity, limited data is available within the field of stroke and in human trials (64, 84). Therefore, fetal MSCs could be good sources of EVs in clinical application.

## Clinial Applications of Extracellular Vesicle-Based Therapy

The effects of EV therapeutics have increasingly been reported in various animal disease/injury models (87). However, only a few clinical studies on the effects of EV therapy have been reported in humans. Kordelas et al. reported a case study, whereby refractory graft-versus-host disease was treated with allogeneic MSC EVs (105). In this report, allogeneic MSCs were cultured in MSC conditioned media and EVs were isolated by the polyethylene glycol (PEG) precipitation method. EVs obtained from 4 × 10^7^ MSCs were administered repetitively four times. Clinical symptoms were improved, and no adverse effects were observed. Katagiri et al. applied allogeneic MSC EVs via local injection for alveolar bone regeneration in eight patients who were diagnosed as needing bone augmentation prior to dental implant placement, which revealed this method was safe and may have great osteogenic potential (106). Lastly, Zhang et al. applied MSC EVs via intravitreal injection in five patients with refractory macular holes (107). All three clinical studies are small case series, and although this data suggests that MSC EVs are safe and may improve patient outcomes, randomization trials are needed to investigate the efficacy and safety of MSC EV therapy. No studies have examined the effects of stem cell-derived EVs in stroke patients. Several phase I/II clinical trials are ongoing to evaluate the application of EVs in cancer patients (108–110).

Considering MSC EVs are the therapeutically active component of MSCs, are non-self-replicating and small sized, the regulatory items required to produce EV fractions for clinical treatment strategies could be less complicated than for MSC therapies. However, compared to MSC therapy, clinical evaluation of EV therapeutics is still at an early stage. Several issues must be considered and need to be solved before the clinical application of EVs, including specific guidelines targeting EV-based therapeutics, characterization, isolation, and storage of EVs, quality control requirements, and *in vivo* analyses of EV. These issues were discussed precisely elsewhere (87, 111, 112), yet the following issues deserve mention in the application of EV for stroke patients.

First, the optimal time and mode of application of EVs should be studied in stroke patients. Most recovery occurs in the first few months following a stroke, with only minor additional measureable improvements occurring thereafter. The levels of chemokines, trophic factors, and related miRNAs increase markedly in the infarcted brain during the acute phase of stroke but decrease over time. Such changes in the brain microenvironment may greatly affect the biodistribution of EVs, as well as the degree of recovery and neurogenesis/angiogenesis after EV therapeutics in stroke patients.

Second, since EVs have many therapeutic components and multiple modes of action, markers for potency and quality control should be chosen carefully and should be measured during the freezing/thawing procedures and storage period. EV therapeutics for stroke patients may differ depending on the time (acute vs. chronic phase) of application. For example, EV cargo components targeting neuroprotection and immunomodulation are needed in patients with acute ischemic stroke, while EV components targeting neurogenesis and angiogenesis are required for neurorestoration in both acute and chronic stroke patients. Differential markers for the potency of EVs (*in vitro* bioassays) may be needed for patients with acute and chronic ischemic stroke. In addition, customized stem cell-EV properties for stroke treatment are needed. Given the heterogeneity of EVs in terms of cargo proteins and RNAs, further studies are needed to increase the therapeutic components of EVs for stroke patients in clinically feasible ways (33, 37, 56, 96, 113).

Lastly, the BBB is formed by the brain capillary endothelium and excludes ~100% of large-molecule neurotherapeutics from the brain and more than 98% of all small-molecule drugs (114). As a result, compared with local application of EVs for topical diseases or other systemic illnesses, stroke patients often require large amounts of stem cells and stem cell-derived EVs. Therefore, selection of culture media and isolation methods are particularly important in EV therapeutics for stroke. Many different cell culture media have been used in the production of EVs, including serum-supplemented media, serum-free media, and EV-free/reduced serum-supplemented media. Because a prior elimination of EVs from fetal bovine serum is crucial, and commercial exosome/EV-depleted serum is expensive and may be imperfect, various methods to deplete EVs are being investigated, such as through the ultrafiltration method (115). In addition, various techniques have currently been used for EV isolation that include (but are not limited to) ultracentrifugation, PEG precipitation, size exclusion chromatography, and tangential-flow filtration. However, each method has advantages and disadvantages, and there is no reliable method for isolation techniques for EVs (112). Recently, GMP-compatible methods for clinical scale production, purification, and isolation of EVs have been introduced (116). Another important issue in improving the therapeutic effects of EV-based therapy in stroke is BBB manipulation, which may enhance endogenous repair mechanisms following stroke, by allowing entry of paracrine factors (e.g., trophic factors and EVs) more easily to the brain (117).

## Conclusion and Future Perspectives

Cell therapy using EVs derived from stem cells could represent a new, clinically feasible, and cell-free paradigm that would avoid cell-related problems. Development of scientific research has just begun in this stem cell-derived EV strategy when compared to that of stem cell therapy. However, MSC-derived EV is rapidly expanding and could be a promising approach for patients with severe stroke, as MSC therapies have already been tested in preclinical and clinical trials and EV-mediated therapy has unique advantages over MSC therapies in stroke patients, in terms of biodistribution (cross the BBB and avoid the first pass effect) and off-the-shelf approaches for acute ischemic stroke.

There have been significant advances in the application of stem cell-derived EVs for human diseases and our understanding of the function and biogenesis of EVs. The efficacy of stem cell-derived EV therapeutics will be improved with advances in our understanding of the biology of stem cells and their EVs, together with advances in techniques to modulate stem cell-derived EV characteristics, including biotechnology and bioengineering. Future studies should focus on our need for more well-designed preclinical studies of EV therapeutics in animal models of stroke. Further studies should particularly focus on biodistribution studies, optimal time/dose/mode of application, and functional outcome measures with neuroimaging data. In addition, the optimal cargo of EVs for EV therapies for stroke patients is unsettled. Moreover, quality management of EVs and establishing standard operating procedures for EV therapeutics are needed, as randomized trials of EV for stroke patients are warranted.

## Author Contributions

OB and EK: study concept and design, acquisition of data, analysis and interpretation of data, drafting/revising the manuscript for content.

### Conflict of Interest Statement

The authors declare that the research was conducted in the absence of any commercial or financial relationships that could be construed as a potential conflict of interest.
